# Striking a balance: triage and crisis intervention models within the pediatric emergency room

**DOI:** 10.3389/fpsyt.2023.1277095

**Published:** 2023-10-18

**Authors:** Nicolas Laporte, Lily Hechtman, Cécile Rousseau, Brian Greenfield

**Affiliations:** ^1^Department of Psychology, Concordia University, Montreal, QC, Canada; ^2^Department of Psychiatry, McGill University, Montreal, QC, Canada; ^3^Department of Psychiatry, Montreal Children's Hospital, Montreal, QC, Canada

**Keywords:** pediatric psychiatry, crisis intervention, triage, emergency room, crisis care, suicidal adolescents, alliance

## 1. Introduction

Upwards of 500,000 pediatric patients visit emergency rooms (ER) annually for psychiatric crises (1), with rates recently increasing (2, 3), necessitating an examination of ER treatment approaches, including triage and crisis interventions. Nurses and pediatricians without specialized psychiatric training often apply a triage approach to such youth's care (4–8), rapidly admitting or discharging them depending on risks of auto- or hetero-aggression. Alternatively, a crisis intervention model emphasizes diagnosis, immediate treatment and orientation to either inpatient and/or outpatient resources on discharge (9–19). This approach is often multimodal (18), including nurses and social workers experienced in pediatric mental health, thus requiring additional resources compared to triage approaches. The triage (4–8) and crisis intervention (9–16) models have mostly been considered in isolation. When considered in tandem (17–20), there is little elaboration on treatment variables (e.g., alliance, the patient and physician's emotional responses and time limitations). This article compares the two models and the interplay of these variables with respect to each. A composite case highlights the differences.

## 2. Triage model

A triage model is time-limited, guided by assessment of suicide acuity as reflected by answers to core questions that expeditiously discern those most at risk of attempting or completing suicide (4–8). This model could begin with a nurse's assessment of the acuity of the suicidal phenomena on a scale of simple ideation to a threat, mild attempt, severe attempt and/or plan, and communicating those results to a pediatrician, who spends 15 min exploring those factors and deciding upon the need for hospitalization (5–8). The brevity of these assessments is suitable for busy ERs, scarce in mental health workers (2, 3). This time-restricted ER triage approach provides only limited information about the origins of the youth's psychiatric disorder, the presence of adversity and treatment options, and opportunities for symptom resolution. With such limitations, a triage approach could yield higher rates of ER return (4, 6). Repeated visits strain the ER, reducing time clinicians allot to youth, diminishing health professional's senses of self-efficacy, thus contributing to apathetic attitudes toward such patients and their families (21), perhaps leading to worse outcomes (22). By contrast, improved training in such youth's care, although rarely available (23), enhances staff's self-efficacy, engagement with and treatment of this population (21). Despite this model's limitations, it may contribute to resolution of negative feelings surrounding the youth's adversity (24), soothed by the security of the ER and an empathetic ear, although potentially offset by the ER's fast pace (25).

## 3. Crisis intervention model

A crisis intervention model would include a multidisciplinary team approach to treating youth and family distress, including diagnosis, treatment planning and community referral (9–19). The first component of this model is identical to the triage model, differing only insofar as the pediatrician subsequently refers the youth and family to a multimodal mental health team, including a psychiatric nurse, psychiatrist and social worker, working together to alleviate distress. This model requires an additional 1–3 h, depending on the needs of the case. Elements central to this intervention could include understanding the underpinnings of the crisis, defining the youth's psychiatric disorder and prescribing psychotherapeutic and/or pharmacologic interventions for its management, identifying and arresting abuse, and developing a treatment plan addressing the underlying disorder and adversity. A mental re-framing of identified core beliefs could reduce the youth's dysphoria, using techniques such as a narrative shift, for example guiding a child from a sense of low self-worth due to a learning disorder to one of normal intelligence, simply learning differently. Such a narrative shift would also help youth perceive themselves as strong and courageous for surviving despite considerable challenges, rather than feeling weak due to past toxic chronic emotional abuse (24). The crisis thus serves as an opportunity to change how youth and caregivers think and feel about themselves and each other, at a time when emotions are heightened and defenses are low, rendering those youth accessible to interventions aimed at reducing their psychological distress. Such an approach can also reduce inpatient admissions (17) and depressive symptomatology (11, 14) and increase adherence to outpatient referrals (9, 10, 13, 16); although findings are mixed (9, 10, 12, 15). Multimodal ER interventions may therefore reduce patient's subsequent emergencies and return visits (18). The repeated reassessment of the child's suicidality using this approach would allow for a more informed perspective on their suitability for hospitalization or outpatient management.

## 4. The development of an alliance

Alliances with pediatric patients are critical in mental health services. They are characterized by the quality of relationship and trust between clinicians and youth/families, strengthened by empathy and active listening (26). Good alliances manifest in a triage model when youth honestly reveal their reasons for ER presentation, maximizing chances that decisions for hospitalization or outpatient management would be best informed. Alliances are critical for an outpatient ER crisis intervention model to enhance the intervention's impact. That outpatient process can be informed by crisis interventions with similar hospitalized high-risk youth. These usually places an emotional burden on youth, family, and health professionals, thus requiring the rapid building of alliance. Strong alliances are associated with treatment adherence (27), reduction in re-admission rates (28) and positive psychotherapy outcomes (29). Alliance building is challenging in any environment but is particularly so in an ER setting, using either model, as the ensuing rapid assessment creates a pressured environment, compromising the clinician's empathetic stance (25). Adverse Childhood Experiences (ACEs) could also developmentally lead to youth's mistrust of caregivers (24), aggression and unpredictability, thus disrupting alliances with healthcare professionals and engendering anxiety and vigilance among them (30).

## 5. The clinician's emotional experience

Occasionally, clinicians experience feelings or thoughts when interacting with youth that surprise them as these were absent prior to the assessment. Such experiences could be named countertransferences. They are often stimulated by the child's early relationships with primary caregivers and then repeated in sessions with the therapist. This is called a transference and has little to do with the clinician themselves. The clinician's unwelcome feelings, absent prior to interactions with youth, probably emanated from the child and/or family (31, 32). Understanding this process can lead to insight into the youth's difficulties and relieve distress. This interplay of emotions, particularly relevant when associated with emotionally dysregulated youth in their pattern of interpersonal difficulties (33, 34), might commonly occur with those exposed to ACEs (24). For example, projective identification occurs when individuals attempt to extricate bad feelings or conflicts by projecting them into another who then acquires the rejected feelings or conflicts as their own (35). Using the clinician's emotional experience and self-awareness, projective identification becomes apparent as it draws the clinician into the youth's emotional orbit beyond simple empathy. Such primitive defenses could be anticipated by ER clinicians when working with youth experiencing intensely negative feelings (e.g., anger, guilt and feelings of incompetence) often resulting from family dysfunction and unattuned and rejecting parents. This interplay of emotions between youth and clinicians, contributing to the therapeutic process, will manifest in both models, but is more elaborated in the crisis intervention model. It would be of benefit to ER staff, although rarely the case (23), to receive mental health training to improve their awareness of such processes. Balint groups could facilitate discussions of these emotionally charged cases and have improved patient-staff communication and empathy and reduced burnout (36).

## 6. Time limitations

The rapid pace and associated ER time constraints must be managed in both triage and crisis intervention approaches (17). Limited time may detract from the validity of observations, negatively impacting decisions to hospitalize/discharge and may impair a perception of a youth's diagnosis and treatment plan. Although the crisis intervention model vs. the triage approach provides more observation time (9–19), neither approach benefits to the same extent from multiple observations over extended periods characterizing an outpatient setting. That setting engenders several evaluations, improving the likelihood of diagnostic and treatment clarity and accuracy.

## 7. Reluctance to accept healthcare

The youth or caregivers' reluctance to accept healthcare may impair an ER clinician's effectiveness. When obliged by family or police to submit to an ER assessment, youth may lack motivation for help-seeking (19). As well, a caregivers' reluctance to submit to evaluation may stem from unwillingness to acknowledge their contribution to the adversity (37). Abusive parents, themselves often victims of childhood abuse, may also be blinded to the adversity they are perpetuating (38). Stopping the adversity can reassure the child of security, contrary to their previous adverse home environment, thus relieving reluctance to change and lessening the youth's suicidality. The child could potentially be discharged to a safer environment, avoiding hospitalization. Adversity resulting in suicidality within a triage context would not likely be addressed prior to hospitalization.

## 8. Case study

Mary was a 14-year-old female, residing with her biological mother and father and 12-year-old sister. She presented to the ER after having told a friend she ingested 15 pills of aspirin 2 days previously while thinking ambivalently about suicide. She had not previously discussed this overdose with anyone nor sought help. The friend informed the mother who brought her to the ER. She had not confided to anyone her two prior attempts within the past 6 months.

### 8.1. Triage model

The clinicians were concerned about her multiple recent ingestions even in the absence of her distress. Without further data characterizing this case in the triage model, the clinicians could legitimately hospitalize this youth awaiting a full multimodal evaluation on an inpatient service.

### 8.2. Crisis intervention model

On further evaluation, the youth revealed that her grades were in the passing range, aside from her customary failures in math. She loved her friends and boyfriend, enjoyed drawing for which she had talent and wanted an art career.

She was easily angered when her father typically called her “stupid” and “lazy” upon failing math exams and when he requested she perform household chores leading to shouting matches. Suicidal feelings would ensue as well as low self-esteem associated with struggles in math, self-perception as an angry young woman and the thought that her parents would be better off without her, as revealed to the psychiatrist. She also noticed frequent distractions during class, fidgeting and having a restless leg.

The youth appreciated an awareness of an underlying ADHD with impulsivity, its treatability and its genetic etiology (39), thereby partially absolving her of guilt related to her angry episodes. Awareness of the factors that leading to suicidal feelings contributed to their resolution. The father acknowledged to the social worker the toxicity of his verbal putdowns, expressing wishes to find alternative ways to communicate with his daughter, thus diminishing her dysphoria.

The youth was informed about strategies to manage future suicidal feelings and was referred to community mental health services. Ideally, an ER follow-up team would provide follow-up services 2–3 days post discharge, absent which the patient's general practitioner/pediatrician was guided in using Guanfacine to manage her irritability and she and her father were referred to dyadic therapy to improve their relationship. Contact with team members 1 month post ER discharge ensured community follow-up, at which point pharmacologic management was confirmed, the irritability had reduced and the relationship with her father and self-esteem had improved while the suicidality had abated.

### 8.3. Relative merits of the two models

The triage model, although ensuring the youth's security through hospitalization, could have benefited from further questioning. For example, the crisis model provided more extensive evaluation of dynamics leading to her suicidality, which were driven by her exposure to adversity and her negative self-esteem reflective of a fast temper. The more elaborated multimodal crisis evaluation also led to identification of resilience factors, understanding of her reasons for distress and their resolution while in the ER, leading to discharge and follow-up care. Although we contrast the two ends of the spectrum between the triage and crisis intervention models in caring for youth in suicidal crisis, evaluations of such patients are usually an amalgam of the two models (20). Their deployment, in a given setting, probably reflects the availability of emergency resources (18).

## 9. Conclusion

Both triage and crisis intervention models share challenges of creating an alliance in a short period while overcoming reluctance to change. The crisis intervention model might provide more opportunities of evoking strong emotions from the youth and family and analogously from the treating clinician. Where resources are available, a crisis intervention model provides the luxury of either a multimodal team and/or a more extensive evaluation while the youth and family are still in the ER, working out suitable treatment plans and potentially reducing the need for hospitalization. A trans-Canadian study is currently underway to measure client satisfaction for youth and families who experience either model. We await that study's results.

## Author contributions

NL: Writing—original draft, Writing—review and editing, Conceptualization. LH: Writing—review and editing. CR: Writing—review and editing. BG: Supervision, Writing—review and editing, Conceptualization.
